# Case report: Cerebrotendinous xanthomatosis treatment follow-up

**DOI:** 10.3389/fneur.2024.1409138

**Published:** 2024-06-17

**Authors:** Karolina Ejsmont-Sowała, Tomasz Książek, Katarzyna Maciorowska-Rosłan, Joanna Rosłan, Agata Czarnowska, Anna Jakubiuk-Tomaszuk, Joanna Tarasiuk, Katarzyna Kapica-Topczewska, Alina Kułakowska

**Affiliations:** ^1^Medical University of Bialystok, Bialystok, Poland; ^2^Department of Neurology, Medical University of Bialystok, Bialystok, Poland; ^3^Department of Pediatric Neurology, Medical University of Bialystok, Bialystok, Poland; ^4^Medical Genetics Unit, Martermed Medical Center, Bialystok, Poland

**Keywords:** CTX, cerebrotendinous xanthomatosis, CYP27A1, chenodeoxycholic acid, CDCA

## Abstract

Xanthomatosis is a genetic disease inherited in an autosomal recessive manner. The specific phenotypic features are associated with patient’s genetic profile. The result of the mutation is disorder of cholesterol synthesis and the accumulation of its precursors in tissues. The characteristic symptoms are progressive cerebellar ataxia, cataract, diarrhea, and the deposition of cholesterol in the tendons. Our objective is to follow-up information to treatment efficacy of 22-year-old patient diagnosed with cerebrotendinous xanthomatosis through 1.5 year observation. In 2012, an 11-year-old patient with a long history of deformed feet and frequent yellowing of the skin, was admitted to the Department of Neurology due to seizures. In 2013, the patient began to suffer from diarrhea, and its frequency was correlated with the concentration of bilirubin in the blood. In the same year cataract was diagnosed. Gradually, the patient starts to complain about progressive difficulties in moving. In 2019, genetic tests confirmed the diagnosis of cerebrotendinous xanthomatosis. Since July 2021, the patient has been treated with chenodeoxycholic acid. The deterioration of patient’s mobility has been significantly inhibited, consequently his quality of life has improved. The presented case report underscores the efficacy of CDCA supplementation in halting the progression of CTX, resulting in marked improvements in the patient’s quality of life.

## 1 Introduction

Cerebrotendinous xanthomatosis (CTX) is an autosomal recessive rare neurometabolic disorder. More than 500 cases have been identified worldwide so far, with a variety of mutations. The characteristic features of the disease are neurological deficits (seizures, atypical parkinsonism, peripheral neuropathy, dementia, cognitive impairment, ataxia, dystonia), juvenile cataract and tendinous xanthoma. According to current reports on the disease, symptoms and progression can differ among individuals. What is more, considerable differences in the phenotype severity can be found in identical twins with CTX (1).

Cerebrotendinous xanthomatosis is a lipid storage disease. Due to the lack of the mitochondrial enzyme sterol 27-hydroxylase, which is involved in bile acid synthesis, the cholesterol synthesis is impaired. What is more, there is an accumulation of its precursors in different tissues (2). Three symptoms predominate in most patients: cataracts and xanthomas which are infantile-onset till young adult-onset, and the progressive spinocerebellar ataxia which usually appears in adult life. Other neurological dysfunctions seen in adults are: dementia, psychiatric disturbances, atypical parkinsonism, seizures, dystonia (3, 4). Even though diarrhea is not included into characteristic triade of the symptoms, mostly it is the first manifestation of the disease (5). Prolonged neonatal jaundice can also be one of the earliest disease symptom. It is classified as a strong indicator for CTX suspicion according to suspicion index for CTX published by Mignarri et al. Another strong indicator, according to the authors, is intellectual disability. It is noticed that even 60% of people with cerebrotendinous xanthomatosis disease may have intellectual disability (6).

The first line treatment is the supplementation of chenodeoxycholic acid (CDCA) by using the exogenous form of it (7). Chenodeoxycholic acid is known as a medicament for dissolution of gallstones (8). The aim of the treatment is to increase the acids, which synthesis is reduced due to the lack of the mitochondrial enzyme sterol 27-hydroxylase. Therefore, the delivered CDCA can give negative feedback on cholesterol 7α-hydroxylase which catalyzes the classical pathway of bile acid synthesis. The products of this reaction are cholestanol and bile alcohols (9).

In 2021 the article about first case series of Polish patients with CTX was published. One of those patients was a 20-year-old male who awaited treatment that time. In Novemeber 2021 the patient started treatment with CDCA (10).

The aim of this study is to evaluate the effect of treatment with chenodeoxycholic acid in a 22-year-old patient diagnosed with Cerebrotendinous xanthomatosis through 1.5 year observation.

## 2 Case description

In 2012, a 11-year-old patient was admitted to the Department of Neurology on account of loss of consciousness and a seizure, accompanied by a protracted history of foot deformities and recurrent jaundice. Commencing in 2013, the patient manifested symptoms of diarrhea, the frequency of which exhibited a correlation with bilirubin concentration. Within the same year, diagnoses of cataracts and Gilbert’s syndrome were established. To date, examinations involving the central nervous system and abdominal imaging have revealed no anomalies. The patient began expressing concerns about progressive mobility challenges due to deformation of the feet. Subsequent to multiple electromyography (EMG) tests, polyneuropathy was conclusively diagnosed, confirming lengthening the latency, slowing down the conduction velocity and extending the F wave latency in the motor fibers of the right tibial and peroneal nerves. A discrete decrease in the M amplitude in motor fibers and a slower response in sensory fibers of the right ulnar nerve. Throughout the course of the ailment, the patient experienced rest and intention tremors, accompanied by manifestations of flaccid syndrome like muscle atrophy or weakened muscle tension and symptoms indicative of pyramidal syndrome in the lower extremities - significantly increased tension, excessive deep reflexes and foot shake. In 2019, genetic testing identified a mutation (c.1184 + 1G > A) present in both alleles of the CYP27A1 gene, thereby confirming the diagnosis of cerebral cholesterosis. Further diagnostic scrutiny revealed the presence of the aforementioned mutation in both parental genetic profiles. Since July 2021, the patient has undergone successful treatment with chenodeoxycholic acid, yielding a cessation in the disease’s progression. This intervention has effectively forestalled the deterioration of mobility, prevented further deformities in the lower limbs, and markedly improved the overall quality of life for the individual.

## 3 Outcome

Prior to commencing treatment, the patient exhibited a bilirubin level of 10.5 mg/dL. Subsequent to one year of therapeutic intervention, specifically in March 2023, a substantial reduction in bilirubin levels was observed, with the metric registering at 2.62. Cholestanol concentrations were also observed to decrease by 30%. Notably, the resolution of diarrhea following the administration of medication represents a pivotal indicator of the patient’s enhanced health. The inhibition of disease progression, combined with a noticeable decrease in bilirubin levels, highlights the encouraging results of treatment, despite the remaining neurological deficit. Nevertheless, the imperative for ongoing medical care and vigilant health monitoring persists to ensure the sustained maintenance of achieved results and the enduring well-being of the patient.

## 4 Treatment

Patients diagnosed with cerebrotendinous xanthomatosis necessitate comprehensive care. It is imperative to provide thorough education to both the patient and their family, fostering informed collaboration for optimal therapeutic outcomes. While acknowledging the current incurability of this genetic disorder, highlighting the potential for well-controlled management becomes essential as it significantly augments the patient’s quality of life and overall satisfaction. The bedrock of treatment lies in pharmacological interventions, with the primary goal being the control of symptoms and the attenuation of disease progression. Medications encompass deoxycholic acid, a naturally occurring substance in the liver, which plays a vital role in fat digestion. By emulsifying and aiding in fat digestion, as well as removing excess cholesterol, it facilitates lipid metabolism regulation and reduces bilirubin levels (11). Enzyme inhibitors, such as simvastatin or lovastatin, targeting lipid accumulation, along with antiepileptic drugs for seizure management, contribute substantially to the therapeutic regimen (12). Nutritional therapy is paramount, with a low-fat, carbohydrate-rich diet recommended to limit lipid intake, particularly saturated fats. Depending on the symptom severity, the incorporation of symptomatic treatment and physical therapy may be introduced to enhance muscle strength and motor function (13). Regular and vigilant patient monitoring emerges as a critical aspect, facilitating the assessment of disease progression and the early detection of complications. This monitoring regimen includes MRI for evaluating cerebral changes and routine ophthalmic examinations (14).

## 5 Discussion

Cerebrotendinous xanthomathosis constitutes a rare genetic disorder associated with the CYP27A1 mutation. It causes lack of the sterol 27-hydroxylase which takes part in the process of metabolism cholesterol finally to cholic acid and CDCA. However, 27-hydroxylase is not the only enzyme involved in cholesterol metabolism. There is also a 7α-hydroxylase due to its activity, 7-α-hydroxycholesterol appears. In physiological situation, producted CDCA gives negative feedback to 7-α-hydroxycholesterol. Lack of the CDCA and reduced amount of cholic acid intensifies convertion of 7-α-hydroxycholesterol to 7α-hydroxy-4-cholesten-3-one and finally to cholestanol and bile acid. Since the amount of cholestanol and bile acids is produced in the organism in larger quantities, it results in the deposition of these substances in lipophilic tissues (10).

On average, the first effects of treatment with CDCA appear after 4 months of therapy, but the effectiveness of therapy is significantly influenced by early diagnosis and early beginning of the treatment (15, 16). During treatment, inhibition and, in some cases, withdrawal of neurological symptoms, improvement of mental condition and reduction of changes in MRI images are observed. After partial remission of symptoms, the effectiveness of treatment may decrease, which indicates that neurological changes are irreversible. This confirms the greatest effectiveness of starting treatment in the early phase of the disease (17). Starting treatment earlier may even result in resolution of neurological changes, while starting treatment at a later stage does not provide such benefits. This is especially observed in CTX-induced parkinsonism, which becomes drug resistant with age (18).

Luyckx E. et al. in a scientific work from 2013. recommends the use of CDCA in combination with a strong HMG-CoA reductase inhibitor. There were presented the effects of treatment of two brothers diagnosed with CTX, who after combined therapy showed significant improvement in cognitive functioning, complete disappearance of peripheral neuropathy and inhibition of the progression of intellectual disorders (19). However, Brlek et al. described case of two brothers whose chenodeoxycholic acid monotherapy was successful. Both brothers reported feeling stronger in their extremities. One of them reported that symptoms associated with diarrhea decreased and the number of stools per day was reduced. The control MRI examination proved no progression of lesion growth in both of them (20). The effectiveness of CDCA monotherapy is also described in an analysis based on the effects of therapy in 43 cases of CTX, in which the average follow-up time was 8 years (21). Before treatment, the average plasma cholestanol concentration was 32 mg/L (normal <5.0 mg/L), which decreased to 6.0 mg/L (−81%). Of the respondents, 63% achieved cholestanol concentration < 5.0 mg/L. In 20% of patients with advanced disease, symptoms continued to worsen. Another data on the effectiveness of CDCA comes from a single-center cohort study from the Netherlands (22). In most patients, the signs and symptoms of the disease, such as diarrhea, polyneuropathy, pyramidal or cerebellar disorders disappeared or improved or stabilized during the study. It was noticeable that epilepsy resolved in patients who suffered with this disease. The CDCA treatment improved the condition of patients with psychiatric and cognitive impairment. The same study determined the safety of CDCA. There were 76 adverse events recorded in 26 of patients. The treatment-related adverse effects were constipation and toxic hepatitis. However, toxic hepatitis was the infant case, which has been already reported (23). Total of 9 serious adverse events were reported in 7 patients. None of them were related to the study drug (22). In pregnant women, it is recommended to stop taking CDCA during pregnancy, however, Duell et al. described two cases of pregnancies during which CTX treatment was continued. After two deliveries complicated by pre-eclampsia, two children were born, one was completely healthy, while the other was diagnosed with periventricular leukomalacia. There was no cause-and-effect relationship between the above-mentioned complications and CDCA treatment (24).

Cerebrotendinous xanthomatosis manifests a wide spectrum of clinical symptoms, stemming from diverse genetic mutations and the accumulation of lipids in different tissues. Noteworthy neurological symptoms characteristic of this malady includes psychomotor slowing, tremors, ataxia, epilepsy, and cognitive impairment (4, 25, 26). However, the molecular mechanisms dictating the emergence of neurological symptoms remain presently elusive and constitute an area of ongoing investigation. The elucidation of these mechanisms holds promise for advancing therapeutic strategies in the future (4).

It has been shown that there is a relationship between the patient’s genetic profile and the occurrence of specific phenotypic features (25, 26). Certain genetic variants, namely c.844 + 1 G- > T, p.N403K, p.R395C, p.R405W, p.T339M, p.T343R have been correlated with the occurrence of epilepsy and dementia (25). Notably, 57% of patients bearing the p.R395C genetic variant have presented with ataxia and Arnold Chiari Malformation type 1. In this subset of individuals, a temporal gap of approximately 7 years has been observed from the onset of diarrhea to the manifestation of the initial neurological symptoms (25). Patients harboring mutations such as c.255 + 1G > T and c.1263 + 1G > A exhibit bilateral cataracts and an elevated incidence of falls, impeding autonomous mobility. The etiology of these manifestations is attributed to compromised lower limb strength. Genetic screening of the parents indicates their status as heterozygous carriers. Patients carrying mutations c.1263 + 1G > A, c.1537C > T (p.R513C), c.1263 + 1G > A, and c.1561dupA (p.K520fs) exhibit a comparable disease course. These individuals display tendon xanthomas and pyramidal signs, including exaggerated deep tendon reflexes and a positive Babinski reflex. However, they do not manifest visual disturbances, diarrhea, or limb deformities. In the last mentioned mutation, c.1263 + 1G > A c.379C > T, present a diverse spectrum of symptoms, encompassing diarrhea and challenges in cooperative efforts during assessments of limb muscle strength. These patients encounter difficulties in both balance and muscle strength, accompanied by neurological symptoms (26).

A table detailing the correlations between specific genetic variants and phenotypic features in CTX has been included in the Supplementary materials (Table 1).

**Table 1:** Correlation between genetic variants and phenotypic features in Cerebrotendinous Xanthomatosis (CTX).

Author	Genotype	Phenotype
Taboada et al. (10)	p.R395C	Ataxia Arnold Chiari type 1 7 years from the onset of diarrhea to the first neurological symptoms
Taboada et al. (15)	p.N403K p.R395C p.R405W p.T339M p.T343R	Epilepsy Dementia.
Jiang et al. (16)	c.255 + 1G > T c.1263 + 1G > A	Cataracts Decreased strength in lower limbs. Subnormal intelligence, diminished speech fluency, and compromised memory
Jiang et al. (16)	c.1263 + 1G > A and c.1537C > T (p.R513C)	Tendon xanthomas Pyramidal signs including deep tendon reflexes Positive Babinski reflex
Jiang et al. (16)	c.1263 + 1G > A and c.1561dupA (p.K520fs)	Tendon xanthomas Pyramidal signs characterized by deep tendon reflexes Positive Babinski reflex
Jiang J, et al. (16)	c.1263 + 1G > A c.379C > T	Diarrhea Gait disturbance Slurring dysarthria Bilateral cataracts Positive pyramidal signs, encompassing hyperactive deep tendon reflexes Positive Babinski signs

## 6 Conclusion

The presented case report underscores the efficacy of CDCA supplementation in halting the progression of CTX, resulting in marked improvements in the patient’s quality of life. The observed reduction in bilirubin levels and resolution of diarrhea serve as pivotal indicators of treatment success. The findings contribute valuable insights into the complexities of CTX pathophysiology, highlighting the intricate interplay between genetic factors and clinical manifestations. Ultimately, ongoing medical care and continuous monitoring remain crucial for sustaining positive treatment outcomes and ensuring the enduring well-being of CTX patients.

## Author contributions

KE-S: Writing – original draft, Writing – review & editing, Conceptualization, Investigation. TK: Writing – original draft, Writing – review & editing, Conceptualization, Investigation. KM-R: Writing – original draft, Writing – review & editing. JR: Writing – original draft, Writing – review & editing. AC: Writing – original draft, Writing – review & editing, Supervision. AJ-T: Writing – original draft, Writing – review & editing. JT: Writing – original draft, Writing – review & editing. KK-T: Writing – original draft, Writing – review & editing. AK: Supervision, Writing – original draft, Writing – review & editing.
