# Supplementary material for: Case report: Cerebrotendinous xanthomatosis treatment follow-up
Source: Front Neurol. 2024 Jun 17;15:1409138. doi: 10.3389/fneur.2024.1409138 (PMC11215067; doi:10.3389/fneur.2024.1409138)
Supplement: Supplementary file 1 [file Table_1.DOCX]

Supplementary Material

**Case report: Cerebrotendinous xanthomatosis treatment follow-up**

Karolina Ejsmont-Sowała*, Tomasz Książek, Katarzyna Maciorowska-Rosłan, Joanna Rosłan, Agata Czarnowska, Anna Jakubiuk-Tomaszuk, Joanna Tarasiuk, Katarzyna Kapica-Topczewska, Alina Kułakowska

*** Correspondence:** Corresponding Author: ejsmont.karolina@gmail.com

# Supplementary Table

| Author | GENOTYPE | PHENOTYPE |
| --- | --- | --- |
| Taboada M, Martínez D, et al.^10^ | p.R395C | - ataxia  - Arnold Chiari type 1  - 7 years from the onset of diarrhea to the first neurological symptoms |
| Taboada M, Martínez D, et al.^15^ | p.N403K  p.R395C  p.R405W  p.T339M  p.T343R | - epilepsy,  - dementia. |
| Jiang J, et al.^16^ | c.255+1G>T  c.1263+1G>A | - cataracts  - decreased strength in lower limbs.  - subnormal intelligence, diminished speech fluency, and compromised memory |
| Jiang J, et al.^16^ | c.1263+1G>A  and  c.1537C>T (p.R513C) | - tendon xanthomas  - pyramidal signs including deep tendon reflexes  - positive Babinski relfex |
| Jiang J, et al.^16^ | c.1263+1G>A  and  c.1561dupA (p.K520fs) | - tendon xanthomas  - pyramidal signs characterized by deep tendon reflexes  - positive Babinski reflex |
| Jiang J, et al.^16^ | c.1263+1G>A  c.379C>T | - diarrhea  - gait disturbance  - slurring dysarthria  - bilateral cataracts  - positive pyramidal signs, encompassing hyperactive deep tendon reflexes  - positive Babinski signs |

**Table 1.** Correlation Between Genetic Variants and Phenotypic Features in Cerebrotendinous Xanthomatosis (CTX).
